# Genome-wide identification of the *TCP* gene family in *Chrysanthemum lavandulifolium* and its homologs expression patterns during flower development in different *Chrysanthemum* species

**DOI:** 10.3389/fpls.2023.1276123

**Published:** 2023-09-29

**Authors:** Xiaoyun Wu, Junzhuo Li, Xiaohui Wen, Qiuling Zhang, Silan Dai

**Affiliations:** ^1^ Beijing Key Laboratory of Ornamental Plants Germplasm Innovation & Molecular Breeding, National Engineering Research Center for Floriculture, Beijing Laboratory of Urban and Rural Ecological Environment, School of Landscape Architecture, Beijing Forestry University, Beijing, China; ^2^ Genomics and Genetic Engineering Laboratory of Ornamental Plants, Department of Horticulture, College of Agriculture and Biotechnology, Zhejiang University, Hangzhou, China

**Keywords:** *TCP* gene family, expression analysis, capitulum development, ray and disc floret, *Chrysanthemum*

## Abstract

TCP proteins, part of the transcription factors specific to plants, are recognized for their involvement in various aspects of plant growth and development. Nevertheless, a thorough investigation of *TCPs* in *Chrysanthemum lavandulifolium*, a prominent ancestral species of cultivated chrysanthemum and an excellent model material for investigating ray floret (RF) and disc floret (DF) development in *Chrysanthemum*, remains unexplored yet. Herein, a comprehensive study was performed to analyze the genome-wide distribution of *TCPs* in *C. lavandulifolium*. In total, 39 *TCPs* in *C. lavandulifolium* were identified, showing uneven distribution on 8 chromosomes. Phylogenetic and gene structural analyses revealed that *ClTCPs* were grouped into classes I and II. The class II genes were subdivided into two subclades, the CIN and CYC/TB1 subclades, with members of each clade having similar conserved motifs and gene structures. Four CIN subclade genes (*ClTCP24*, *ClTCP25*, *ClTCP26*, and *ClTCP27*) contained the potential miR319 target sites. Promoter analysis revealed that *ClTCPs* had numerous *cis*-regulatory elements associated with phytohormone responses, stress responses, and plant growth/development. The expression patterns of *ClTCPs* during capitulum development and in two different florets were determined using RNA-seq and qRT-PCR. The expression levels of *TCP*s varied in six development stages of capitula; 25 out of the 36 *TCPs* genes were specifically expressed in flowers. Additionally, we identified six key *ClCYC2* genes, which belong to the class II TCP subclade, with markedly upregulated expression in RFs compared with DFs, and these genes exhibited similar expression patterns in the two florets of *Chrysanthemum* species. It is speculated that they may be responsible for RFs and DFs development. Subcellular localization and transactivation activity analyses of six candidate genes demonstrated that all of them were localized in the nucleus, while three exhibited self-activation activities. This research provided a better understanding of *TCPs* in *C. lavandulifolium* and laid a foundation for unraveling the mechanism by which important *TCPs* involved in the capitulum development.

## 1 Introduction

*Chrysanthemum* ×*morifolium* Ramat., a member of the Asteraceae family (Anthemideae), has been under cultivation in China for more than 3,000 years because of its high economic and ornamental values. These species have attractive ornamental traits with colorful and various types of flowers. *C. ×morifolium* exhibits a prominent ornamental trait in the form of a typical radiate capitulum, commonly referred to as a pseudanthium. This structure comprises central disc florets (DFs) and peripheral ray florets (RFs), making it one of the most notable features of the plant (Bello et al., 2013; Ibanez et al., 2017). Moreover, DFs and RFs display distinct variations concerning gender differentiation, tissue structure, organ fusion, and pigment distribution (Stevens et al., 2001; Li et al., 2010; Bello et al., 2013; Wen et al., 2019b). Investigations on transcriptomics and gene functions have provided many candidate genes potentially responsible for the differences between the two floret types (Broholm et al., 2008; Chapman et al., 2012; Tahtiharju et al., 2012; Juntheikki-Palovaara et al., 2014; Huang et al., 2016; Liu et al., 2016; Zhao et al., 2016; Bello et al., 2017; Zhang et al., 2017; Chen et al., 2018; Wen et al., 2019a; Shen et al., 2021). However, the mechanisms underlying the development of the two floret types are still uncertain.

Transcription factors (TFs) play vital roles in flower development and their temporal and spatial expression differences are usually closely related to flower morphology (Doebley, 1993; Doebley and Lukens, 1998). *TCPs* are responsible for a variety of plant growth processes, including shoot branching (Aguilar-Martinez et al., 2007; Martin-Trillo et al., 2011), plant height regulation (Daviere et al., 2014; Shi et al., 2016), leaf development (Palatnik et al., 2003; Crawford et al., 2004), flower development (Luo et al., 1996; Feng et al., 2006; Tahtiharju et al., 2012; Garces et al., 2016; Huang et al., 2016; Bello et al., 2017; Chen et al., 2018; Wen et al., 2019a; Shen et al., 2021), fruit (Leng et al., 2019) and seed development (Takeda et al., 2006; Nag et al., 2009), and other biological and abiotic stress response (Viola et al., 2016; Guan et al., 2017). Conducting a genome-wide analysis of *TCPs* in *C. lavandulifolium* holds significant importance in gaining deeper insights into the molecular mechanisms regulating flower development.

TCP proteins are plant-specific TFs responsible for several processes of plant growth/development by modulating cell proliferation and differentiation (Cubas et al., 1999; Lopez et al., 2015). These proteins are characterized by a TCP domain, containing a conserved 59-residue-long basic helix-loop-helix (bHLH) structure at the N-terminus. This domain is known for its roles in DNA binding, protein-protein interaction, and facilitating nuclear localization of the protein (Danisman et al., 2013). TCPs were classified as two subfamilies, distinguishing them based on dissimilarities in their TCP domains: class I (known as PCF or TCP-P) and class II (or referred to as TCP-C). The most obvious distinction between the two subfamilies is that class I members lack 4 amino acids in the TCP domain (Martin-Trillo and Cubas, 2010). The class II TCPs are subdivided into 2 subclades (CYC/TB1 and CIN) in accordance with the differences between the TCP domains. The former subclade has eight members in *Arabidopsis*, out of which five are modulated by miR319 (Palatnik et al., 2003). Some class II members comprise an arginine-rich motif (R domain) that is responsible for the facilitation of protein-protein interaction (Cubas et al., 1999; Martin-Trillo and Cubas, 2010). Phylogenetic analysis indicates that the CYC/TB1 clade is subdivided into 3 types of genes that expanded through duplication within the group: CYC1, 2, and 3. Taking into account that two classes of TCP proteins possess distinct yet partially overlapping DNA binding sites with the consensus sequence GGNCCC (Kosugi and Ohashi, 2002), previous research has indicated that class I and II TCP proteins are responsible for synergistic and antagonistic biological interactions (Li, 2015), which usually occurs between the same class of TCP transcription factors.

Class I TCP family members enhance cell proliferation and growth, however class II members suppress these processes (Herve et al., 2009; Li et al., 2019). Studies have shown that some class I TCP TFs have been found to participate in various hormone-signaling pathways and play crucial roles in regulating inflorescence stem elongation (Daviere et al., 2014), gynoecium development (Lucero et al., 2015), seed germination (Resentini et al., 2015), filament elongation of stamen (Gastaldi et al., 2020) and flowering (Lucero et al., 2017) in *Arabidopsis*. Compared to class I members, the functions of the majority of class II *TCP*s have been extensively studied and understood. For instance, *Arabidopsis* CIN-TCPs are associated with the regulation of flowering time and floral organ development (Kubota et al., 2017; Li et al., 2019), and recent advances in the function and regulation of CIN-like *TCPs* are well described (Lan and Qin, 2020). It is well known that *TB1* participates in the determination of maize axillary meristem fate (Doebley et al., 1997) and *CYC* controls the asymmetry of petals in *Antirrhinum* flower (Luo et al., 1996). In *Arabidopsis*, *AtTCP12* and *AtTCP18* are two homologs of *TB1* that are responsible for inhibiting bud outgrowth (Aguilar-Martinez et al., 2007). Moreover, AtTCP18 is responsible for the suppression of the early floral transition in axillary meristems by binding with florigen proteins (Niwa et al., 2013). *AtTCP1* is the homologous gene of *CYC*, which affects plant growth/development by regulating the expression levels of *DWARF4*, a brassinosteroid (BR) biosynthesis gene (Guo et al., 2010), and also affects leaf elongation growth (Koyama et al., 2010). Additionally, *CYC*-like genes have been shown to regulate floral symmetry in many species, such as *Primulina heterotricha* (Gao et al., 2008; Yang et al., 2012), *Torenia fournieri* (Su et al., 2017), *Saintpaulia ionantha* (Hsu et al., 2018), *Gerbera hybrida* (Elomaa et al., 2018; Zhao et al., 2020), *Chrysanthemum* (Huang et al., 2016; Chen et al., 2018; Shen et al., 2021) and so on.

Recently, *TCPs* have been discovered in various plant species, and the number of *TCPs* varies among different plants. For example, *Arabidopsis*, *Solanum lycopersicum*, *Oryza sativa*, *Populus euphratica*, *P. trichocarpa*, *Malus domestica* and *Prunus mume* have 24, 30, 28, 33, 36, 52 and 19 *TCPs*, respectively (Martin-Trillo and Cubas, 2010; Parapunova et al., 2014; Xu et al., 2014; Ma et al., 2016; Zhou et al., 2016). However, the *TCP* gene family in *C. lavandulifolium* remains relatively unexplored, despite its significance as an origin species of cultivated chrysanthemum (Dai et al., 2002). This plant also serves as an excellent model material for investigating the development of RFs and DFs in *Chrysanthemum*. Recognizing the crucial roles of *TCPs* in plant growth/development, we undertook an extensive examination of the *TCP* gene family in *C. lavandulifolium*. Herein, we identified 39 *TCPs* from the *C. lavandulifolium* genome and executed a comprehensive analysis, including chromosomal position, phylogenetic relationship, conserved motifs, gene structures, and miRNA target sites. To clarify their functions in flower development, we further analyzed the expression patterns of *ClTCPs* during capitulum development and in two different florets, as well as the key *ClTCPs* were tested by qPCR in *Chrysanthemum* species. Additionally, the subcellular localization characteristics and the self-activation activity in yeast of key TCP proteins were also examined. This study not only provided a basis for a comprehensive understanding of *TCPs* in *Chrysanthemum*, but also laid a foundation for revealing the mechanism of *TCP*s regulating the capitulum development.

## 2 Materials and methods

### 2.1 Plant materials and growth conditions

The *C. lavandulifolium* G1 line, a diploid relative wild species for *C.* ×*morifolium* Ramat., which has achieved genomic sequence (Wen et al., 2022), was the main material for gene family analysis and screening key *ClTCPs*. Other *Chrysanthemum* samples, including *C. indicum* (Ci), *C. vesticum* (CVW), *C*. ×*morifolium* ‘C27’, ‘D91’, ‘G70’, ‘T3’, and *C. aromaticum* (SN) were chosen to detect the expression profiles of target genes in DFs and RFs. All plant materials were planted and managed under conventional field conditions in the chrysanthemum germplasm nursery at Beijing Forestry University (Beijing, China).

### 2.2 Identification of *TCPs* in *C. lavandulifolium*

Genome sequences were obtained from the *C. lavandulifolium* database (Wen et al., 2022). To determine the *TCPs* in *C. lavandulifolium*, we used the *Arabidopsis* Information Resource (TAIR) database (http://www.arabidopsis.org) to download all AtTCP protein sequences as reference sequences and screened the *C. lavandulifolium* genome database using local BLAST comparisons. All ClTCP protein sequences were verified by the Swissprot database (https://www.sib.swiss/swiss-prot). We removed repeated sequences using Excel and further analyzed the obtained non-redundant ClTCP sequences. Subsequently, we calculated the molecular weights (MW) and isoelectric points (pI) of ClTCP proteins using the ExPasy website (https://web.expasy.org/protparam/) and predicted the subcellular localization of the ClTCPs through WoLF PSORT (http://www.genscript.com/psort/wolf_psort.html).

### 2.3 Phylogenetic analysis, conserved motif and gene structure identification

To explore the phylogenetic relationships of *TCPs* between *C. lavandulifolium* and *Arabidopsis*, we used MAFFT v7 (https://mafft.cbrc.jp/alignment/server/index.html) to perform multiple sequence alignments and subsequently used MEGA X software to develop a phylogenetic tree according to the multiple alignments data using the Maximum Likelihood (ML) approach with 1000 replicates.

Conserved motifs of TCP proteins were discovered and evaluated by MEME (https://meme-suite.org/) (parameter settings: max. number of motifs 10; max. width of motifs 50; min. width of motifs 6), and the SMART (http://smart.embl-heidelberg.de/) was employed to annotate the identified motifs. The TBtools toolkit (Chen et al., 2020) was used to assess the gene structures with the corresponding DNA sequences and CDSs at default parameter settings.

### 2.4 Chromosomal position and miR319 target site prediction

Based on positional information from the *C. lavandulifolium* genome, locations of *TCPs* on the *C. lavandulifolium* chromosomes were determined using the TBtools toolkit. To predict target sites for miR319, we used the psRNATarget online application (https://www.zhaolab.org/psRNATarget/analysis?function=3) to analyze *ClTCPs* nucleotide sequences.

### 2.5 *In silico* promoter analysis

The 2,000 bp upstream of the *ClTCPs* coding regions were extracted from *C. lavandulifolium* genome data, which was regarded as the promoter sequences. We used the PlantCARE tool (http://bioinformatics.psb.ugent.be/webtools/plantcare/html/) for the identification of the putative *cis*-acting elements of *ClTCP* promoters.

### 2.6 Expression patterns of *ClTCPs* in various development stages and two different florets

We obtained the expression patterns of *ClTCPs* in six development stages of capitula (S1, S2, S5, S6, S9, and S10) and two different florets (R1, D1, R2, D2) from the previously reported RNA-seq data (Wen et al., 2019a; Wen et al., 2022). Data normalization was conducted according to the average expression value of each gene across all studied samples. Genes and their expression patterns were systematically grouped according to the average Person’s metric. Finally, the heatmap of *ClTCPs* was constructed with TBtools (Chen et al., 2020).

### 2.7 Expression analysis of *ClCYC2* genes by quantitative real-time PCR

The qRT-PCR was conducted to analyze the expression patterns of key differential *ClTCPs* in various *Chrysanthemum* species. Total RNA was isolated from the DFs and RFs at two development stages (R1, D1, R2, D2) using the RNAprep-Pure-Plant-Kit (Tiangen, China) and used for cDNA synthesis with the FastKing-RT-Kit (Tiangen, China), qRT-PCR analysis was conducted on a CFX96-real-time-system (Bio-Rad, USA) using the SYBR-Premix-Ex-Taq-kit (Takara, Japan). The primer pairs are presented in **Supplementary Table 1**. We used *ClSAND* as a reference gene (Qi et al., 2016; Pu et al., 2020; Lu, 2022).

### 2.8 Subcellular localization of ClCYC2 proteins

The cDNA of the *ClCYC2a*, *ClCYC2b*, *ClCYC2c*, *ClCYC2d*, *ClCYC2e*, and *ClCYC2f* without stop codons were amplified with the primer pairs (**Supplementary Table 2**) and recombined into pBI121-eGFP to generate six fusion constructs. *Agrobacterium tumefaciens* strain GV3101 was employed to introduce the recombinants and pBI121-eGFP (used as a control) into the leaves of *Nicotiana benthamiana* through infiltration. After 48 hours, the expression of GFP was examined under a confocal laser scanning microscope (Leica-TCS-SP8, Wetzlar, Germany).

### 2.9 Analysis of transactivation activity in yeast

To assess the transactivational activity of six ClCYC2 proteins in yeast, we employed the pGBKT7 vector (BD, Clontech, United States) for the experiments. PCR amplification of the entire *ClCYC2* open reading frames was performed with the gene-specific primers pairs (**Supplementary Table 3**). These amplicons were then independently recombined into the pGBKT7 vector. Following this, we co-transformed the pGBKT7-ClCYC2 recombinant vectors, pGBKT7-53, and pGBKT7-lam, along with pGADT7 (AD, Clontech, United States) empty vectors, into the Y2HGold strain. The transformed strains were then grown in SD/-Leu-Trp and SD/-Ade-His-Leu-Trp media, and then incubated at 30°C for 3-5 days.

## 3 Results

### 3.1 Identification and chromosomal location of *TCP*s in *C. lavandulifolium*

**Figure 1:**
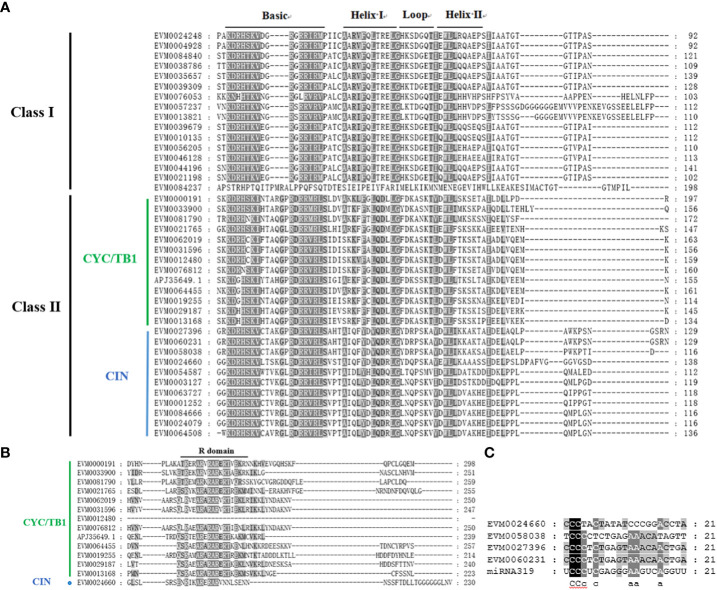
Multiple sequence alignments of TCP proteins in *C. lavandulifolium* and analysis of the potential miR319-targeted sites. **(A)** TCP domain alignment for the ClTCP proteins. The conserved amino acids were shaded in grey, and the bHLH regions were marked. **(B)** R domain alignment of the class II subfamily ClTCP members. Multiple sequence alignments were performed with Genedoc. **(C)** Alignment of potential target sites for miR319 (aligned in reverse). Target sites were taken from the coding sequences of *ClTCP*s, and the mature miR319 sequence was obtained from miRBase (http://www.mirbase.org/).

The *C. lavandulifolium* genome database offers the references for the genome-wide detection of its candidate genes (Wen et al., 2022). In order to identify the *TCPs* in *C. lavandulifolium*, we performed a BLAST search against the *C. lavandulifolium* genome database using all the *Arabidopsis* TCP protein sequences as the query. A total of 39 sequences were obtained. Including one sequence isolated from the transcriptome (Wen et al., 2022), there were a total of 40 sequences obtained, which confirmed the occurrence of the TCP domain using the Swissprot database, indicating there were at least 40 *TCPs* in *C. lavandulifolium*. Among these genes, 14 *TCPs* with R domain were identified (**Figure 1**). In addition, The TCP proteins displayed diversity in their characteristics, including variations in length, MW, and pI. The protein lengths varied between 138 and 435 amino acids, the MW ranged from 15.14 to 46.37 kDa, and the range of pI was 5.51-10.46 pH. According to WoLF PSORT predictions, the majority of ClTCP proteins showed nuclear localization, while a few were found in the cytoplasm and chloroplast (**Table 1**).

**Table 1:** Characteristics of the *TCP* gene family members in *C. lavandulifolium*.

Name	Gene ID	Length (aa)	MW (Da)	pI (pH)	Class	Predicted subcellular location
*ClTCP1*	EVM0035657.1	385	41982.31	8.66	PCF	Nucleus
*ClTCP2*	EVM0039309.1	371	39803.76	6.50		Nucleus
*ClTCP3*	EVM0038786.1	241	26626.14	6.34		Nucleus
*ClTCP4*	EVM0084840.1	435	46368.91	6.36		Nucleus
*ClTCP5*	EVM0004928.1	258	27127.22	9.16		Nucleus
*ClTCP6*	EVM0024248.1	258	27127.22	9.16		Nucleus
*ClTCP7*	EVM0044196.1	312	34008.76	8.87		Nucleus
*ClTCP8*	EVM0010135.1	249	26802.77	10.46		Nucleus
*ClTCP9*	EVM0039679.1	262	28290.40	9.99		Nucleus
*ClTCP10*	EVM0021198.1	273	29216.46	8.01		Nucleus
*ClTCP11*	EVM0046128.1	300	32206.05	6.41		Nucleus
*ClTCP12*	EVM0056205.1	253	27296.60	5.99		Nucleus
*ClTCP13*	EVM0084237.1	283	32330.77	9.25		Chloroplast
*ClTCP14*	EVM0076053.1	169	18700.13	6.33		Nucleus
*ClTCP15*	EVM0013821.1	138	15137.13	5.57		Cytosol
*ClTCP16*	EVM0057237.1	140	15314.30	5.51		Cytosol
*ClTCP17*	EVM0024079.1	289	32496.57	8.58	CIN-like	Nucleus
*ClTCP18*	EVM0084666.1	290	32540.63	9.12		Nucleus
*ClTCP19*	EVM0064508.1	309	34849.25	9.11		Nucleus
*ClTCP20*	EVM0001252.1	343	38431.69	6.26		Nucleus
*ClTCP21*	EVM0063727.1	343	38431.69	6.26		Nucleus
*ClTCP22*	EVM0003127.1	339	37831.17	6.36		Nucleus
*ClTCP23*	EVM0054587.1	269	30497.28	6.86		Nucleus
*ClTCP24*	EVM0024660.1	417	45287.43	9.26		Nucleus
*ClTCP25*	EVM0058038.1	379	41547.33	5.95		Nucleus
*ClTCP26*	EVM0027396.1	397	43282.89	6.11		Nucleus
*ClTCP27*	EVM0060231.1	398	43394.12	6.11		Nucleus
*ClCYC1a*	EVM0033900.1	334	38223.71	9.16	CYC/TB1-like	Nucleus
*ClCYC1b*	EVM0081790.1	304	35014.35	9.06		Nucleus
*ClCYC1c*	EVM0000191.1	384	43558.87	5.91		Nucleus
*ClCYC2a-1*	EVM0031596.1	273	31108.24	9.64		Nucleus
*ClCYC2a-2*	EVM0062019.1	274	31162.16	9.62		Nucleus
*ClCYC2a-3*	EVM0012480.1	212	24124.25	9.63		Nucleus
*ClCYC2a-4*	EVM0076812.1	271	30793.75	9.65		Nucleus
*ClCYC2b*	KX161380.1	257	29342.01	9.29		Nucleus
*ClCYC2c*	EVM0019255.1	245	27694.12	9.23		Nucleus
*ClCYC2d*	EVM0064455.1	322	37257.71	9.02		Nucleus
*ClCYC2e*	EVM0029187.1	304	34803.60	6.10		Nucleus
*ClCYC2f*	EVM0013168.1	287	32715.67	8.90		Nucleus
*ClCYC3*	EVM0021765.1	348	39990.11	7.77		Nucleus

**Figure 2:**
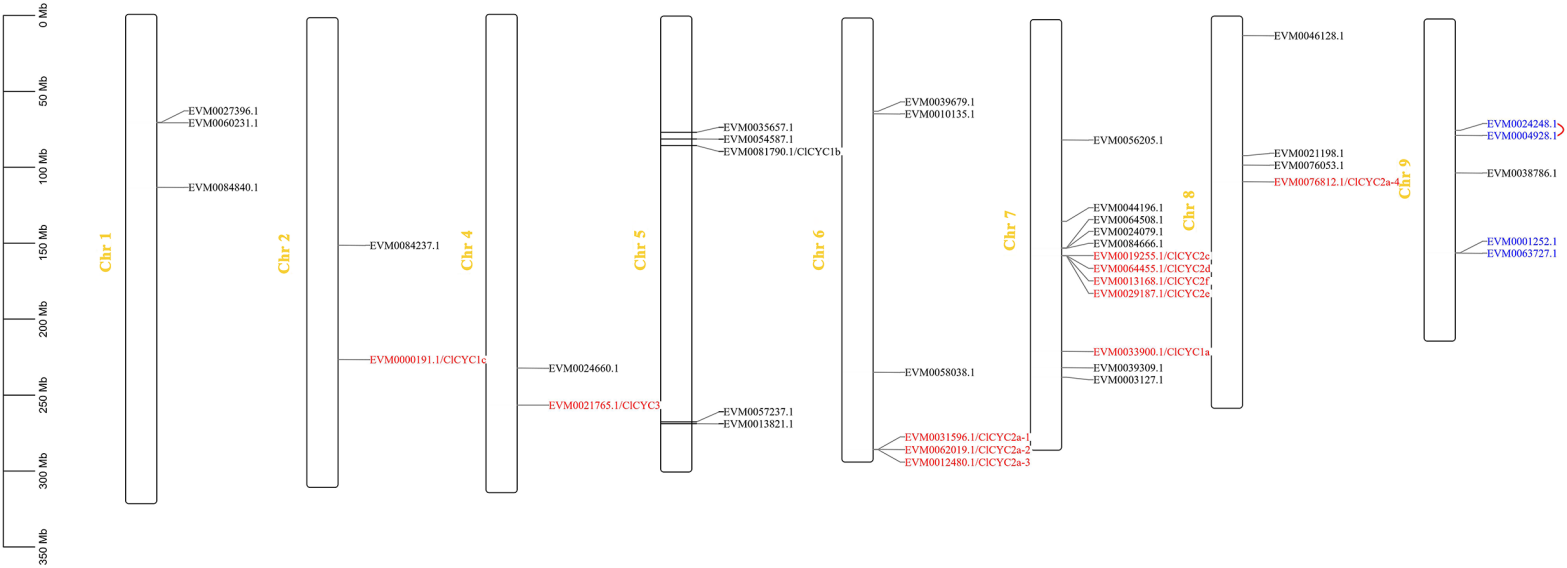
Chromosomal positions of *TCP*s on *C. lavandulifolium* chromosomes. The scale shows a chromosomal distance of 50 Mb. All 39 *ClTCPs* were localized to the chromosomes. Segmentally duplicated genes (*EVM0004928.1_ClTCP5* and *EVM0024248.1_ClTCP6*, *EVM0001252.1_ClTCP20* and *EVM063727.1_ClTCP21*) were marked with blue letters. Eleven *ClTCPs* belong to CYC/TB1 subclade, which are highlighted in red and located on the chromosomes.

We located a total of 39 *ClTCPs* on chromosomes (**Figure 2**), but they were unevenly distributed across the *C. lavandulifolium* genome. There were no genes identified on Chr 3. Among them, members of the CYC/TB1 subfamily were mapped to Chr 2, 4, 6, 7, and 8, respectively. Especially, four *ClTCPs* (*ClCYC2c*, *ClCYC2d*, *ClCYC2e*, and *ClCYC2f*) which belong to the CYC2 subclade formed a gene cluster on Chr 7, and subfunctionalization might have occurred in these genes throughout the evolutionary process of the *C. lavandulifolium* genome (Wen et al., 2022). While the multi-copy genes of *ClCYC2a-1*, *ClCYC2a-2*, and *ClCYC2a-3* formed a gene cluster on Chr 6. Two segmentally duplicated gene pairs (*ClTCP5* and *ClTCP6*, *ClTCP20* and *ClTCP21*) were located on Chr 9.

### 3.2 Phylogenetic analyses and classification of *ClTCP*s

**Figure 3:**
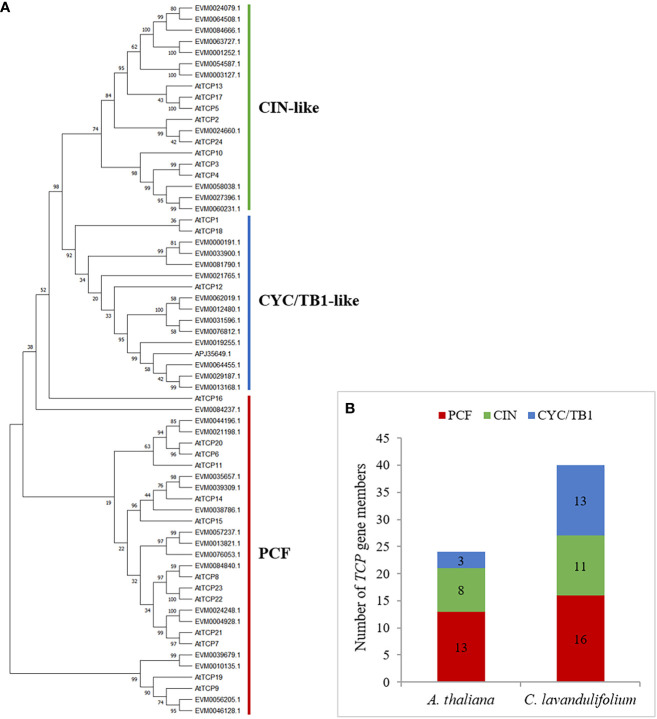
Phylogenetic and statistical analyses of TCP members from *C. lavandulifolium* and *Arabidopsis*. **(A)** The tree was built using the Maximum Likelihood (ML) method with 1000 replicates by MEGA X. Multiple sequence alignments of ClTCPs was performed using MAFFT v7. The bootstrap value was indicated by the number above the branches and values less than 50 would not be displayed. Red, green and blue lines indicate the PCF, CIN and CYC/TB1 subclades, respectively. **(B)** Statistical analysis of TCP members from *C. lavandulifolium* and *A. thaliana*.

To investigate the phylogenetic and evolutionary relationships between the *C. lavandulifolium TCPs* and *Arabidopsis TCPs*, a phylogenetic tree was established using the complete sequences of 40 TCP proteins from *C. lavandulifolium* and 24 TCP proteins from *Arabidopsis*. According to the previous classification of *TCPs* in *Arabidopsis*, phylogenetic analysis and multiple sequence alignments all showed that ClTCP proteins were divided into two categories: class I and II, which contain 16 and 24 genes, respectively (**Figures 1A**, **3**). It was clear that class II proteins possess an additional four amino acids in the basic domain compared to class I members (**Figure 3A**). The R domains were identified at the C-terminus of all ClTCP proteins belonging to class II CYC/TB1, as well as one member from the CIN class (**Figure 3B**). The phylogenetic analysis indicated that class II was subdivided into two subclades, CIN (11) and CYC/TB1 (13). Then CYC/TB1 is further divided into three branches: CYC1, 2 and 3. In *C. lavandulifolium*, the numbers of CYC1, CYC2 and CYC3 protein members were 3, 9 and 1, respectively. Compared with *Arabidopsis*, the Class II *TCPs* in *C. lavandulifolium* were significantly expanded, especially the CYC/TB1 subclade with 13 members, and 9 *TCPs* belonging to the CYC2 branch. Studies on the *CYC2*-like genes shown that they regulate the floret morphology in *G. hybrida*, *Helianthus annuus*, *Senecio vulgaris* and *C.* ×*morifolium* (Tahtiharju et al., 2012; Garces et al., 2016; Huang et al., 2016). The phylogenetic tree suggested that particular genes could have experienced evolution specific to each species.

Additionally, four CIN subclade genes (*ClTCP24*, *ClTCP25*, *ClTCP26*, and *ClTCP27*) contained miR319 target sites and showed a high degree of similarity with miR319-targeted *TCPs* in *Arabidopsis* (**Figure 1C**).

### 3.3 Conserved motif and gene structure identification

**Figure 4:**
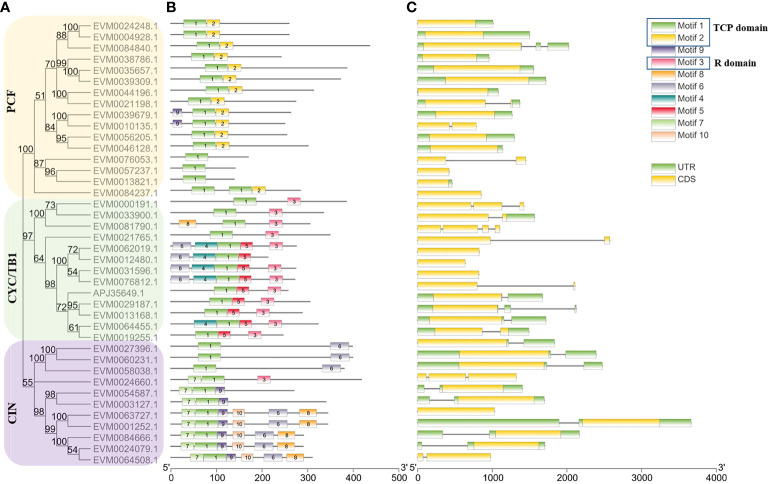
Conserved motif distributions and gene structure of *ClTCP*s. **(A)** The tree was generated based on the sequences of ClTCP proteins. The yellow, purple and green rectangles indicated the clustering of genes into the PCF, CIN and CYC/TB1 subclades, respectively. **(B)** Conserved motifs in *C. lavandulifolium* TCP members were identified by MEME tools. Each motif is indicated by a rectangular box of a different color and numbered from 1 to 10. **(C)** Gene structure analysis of *ClTCP*s. The coding sequence (CDS), untranslated region (UTRs) and introns are indicated by yellow boxes, green boxes and blank lines, respectively.

The conserved motif and gene structures of *ClTCPs* were examined to reveal the structural and evolutionary characteristics of the TCP proteins in *C. lavandulifolium.* By utilizing the complete sequences of the ClTCP proteins, a phylogenetic tree was constructed, effectively categorizing the ClTCP proteins into three distinct subgroups (**Figure 4A**). Ten conserved motifs were identified by the MEME online tool to gain a further understanding of the variety of motif compositions among ClTCPs. The results indicated that all ClTCP proteins had highly conserved TCP domains (motif 1). Almost all of the CYC/TB1 subclade proteins and one CIN protein hit the conserved R domains (motif 3) (**Figure 4B**). In contrast, most CIN proteins contained the N-terminal TCP domains of motif 7. Furthermore, motif 2 was only found in PCF, supporting previous research (Wei et al., 2016; Lei et al., 2017) that certain motifs present in a specific subgroup can play particular roles in those genes.

The intron-exon organization of nearly all *ClTCPs* was relatively conserved, 28 of the 39 *ClTCPs* had no introns, while the remaining 11 genes had one to three introns. Except for *ClTCP8*, *ClTCP10*, and *ClTCP14*, we discovered that no TCP-P (PCF) type genes included introns. Sequences of TCP-C genes in *C. lavandulifolium* had more introns compared to TCP-P genes. Within the TCP-C genes, five of them contained an intron, two contained two introns, and one contained three introns. In summary, the intron-exon distribution patterns of most *ClTCPs* within the same subfamily are similar, which reinforces the subclade classification and supports the understanding of their evolutionary relationships (**Figure 4C**).

### 3.4 Analysis of promoter *cis*-regulatory elements in *ClTCP*s

**Figure 5:**
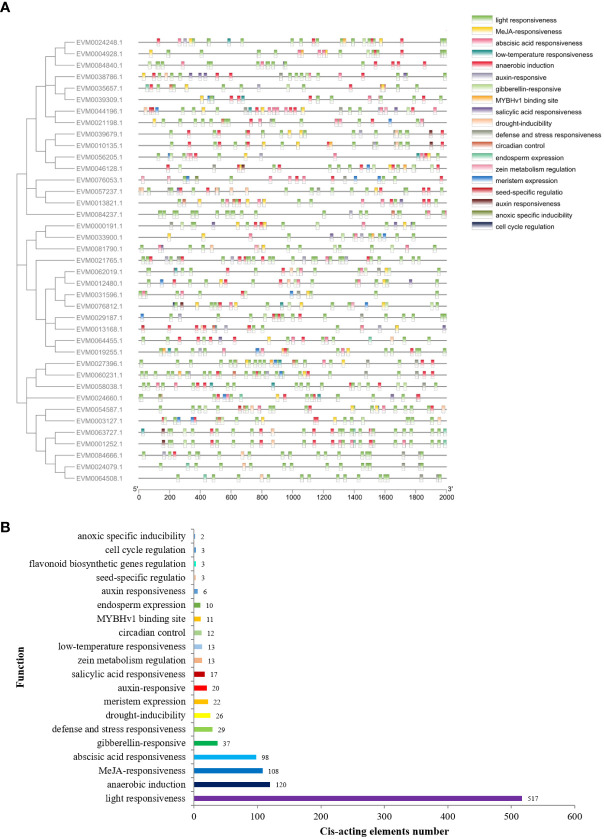
*Cis*-regulator elements analysis of *ClTCP* promoters. **(A)** Distribution of *cis*-regulator elements in the promoter region (2 kb upstream of the transcription start site) of *ClTCP*s. **(B)** Statistical analysis on the number and functional type of *cis*-regulatory elements in all *ClTCP* promoters in *C. lavandulifolium*.

The *cis*-regulatory elements in the promoter sequences were examined to enhance the comprehension of gene functions and regulatory mechanisms of *ClTCPs*. The *ClTCP* gene promoter regions, which are a 2-kb segment of genomic DNA located upstream of the translation start site, were submitted to the PlantCARE database. Numerous *cis*-acting elements associated with phytohormone responses, stress responses, and plant growth/development were identified apart from the basic CAAT and TATA boxes (**Figure 5**; **Supplementary Table 4**). Among them, the largest number of elements were the light-responsive, MeJA-responsive and abscisic acid-responsive elements. Additionally, the elements of auxin-responsive, stress-responsive, cell cycle regulation and meristem regulation were also found in their promoter regions of *ClTCPs* (**Figure 5**).

A kind of *cis*-regulatory elements associated with endosperm expression (GCN4_motif) was determined in the promoter region of 8 *ClTCPs*, and a kind of elements associated with meristem expression (CAT-box) was determined in 18 *ClTCPs* (**Supplementary Table 4**). The circadian control element (circadian) and cell cycle regulation element (MSA-like) were observed in 10 and 3 *ClTCPs*, respectively. Furthermore, the flavonoid biosynthetic genes regulation (MBSI), and seed-specific regulation element (RY-element) were also detected in the *ClTCPs* promoter regions (**Figure 5**; **Supplementary Table 4**).

In hormone-associated *cis*-acting elements, the MeJA-responsive element (CGTCA and TGACG motifs), the ABA-responsive element (ABRE) and the gibberellin-responsive element (TATC-box, GARE-motif and P-box) were found in the promoter regions of 28, 37 and 21 *ClTCPs*, respectively. The salicylic acid (SARE and TCA-element) and auxin-responsive element (TGA-element, AuxRE and AuxRR-core) were discovered in 12 and 18 *ClTCPs*, respectively. The promoter region of *ClTCPs* had an abundance of hormone-responsive elements, suggesting that hormones may be crucial regulators of plant growth/development. Moreover, *cis*-elements associated with stress, such as low-temperature (LTR) responsiveness, drought-inducibility (MBS) and anaerobic induction (ARE) elements were also determined in the promoter regions of 10, 18 and 34 *ClTCPs*, respectively (**Figure 5**; **Supplementary Table 4**).

### 3.5 Expression patterns of *ClTCPs* during capitulum development and in two different florets

The *TCPs* play vital roles in multiple progress during plant development. The previously reported RNA-seq data (Wen et al., 2019a; Wen et al., 2022) were applied to investigate the expression patterns of *ClTCPs* in six developmental stages of capitula (S1, S2, S5, S6, S9, and S10) and two different florets (R1, D1, R2, D2), to explore their functions in flower development. Through searching and blast alignment, a total of 40 *TCP* sequences were obtained, and the genes whose expression level FPKM value was 0 in all samples were removed. Finally, 36 and 35 *ClTCPs* corresponding to the two transcriptomes were used for drawing the expression heatmap (**Supplementary Tables 5, 6**).

**Figure 6:**
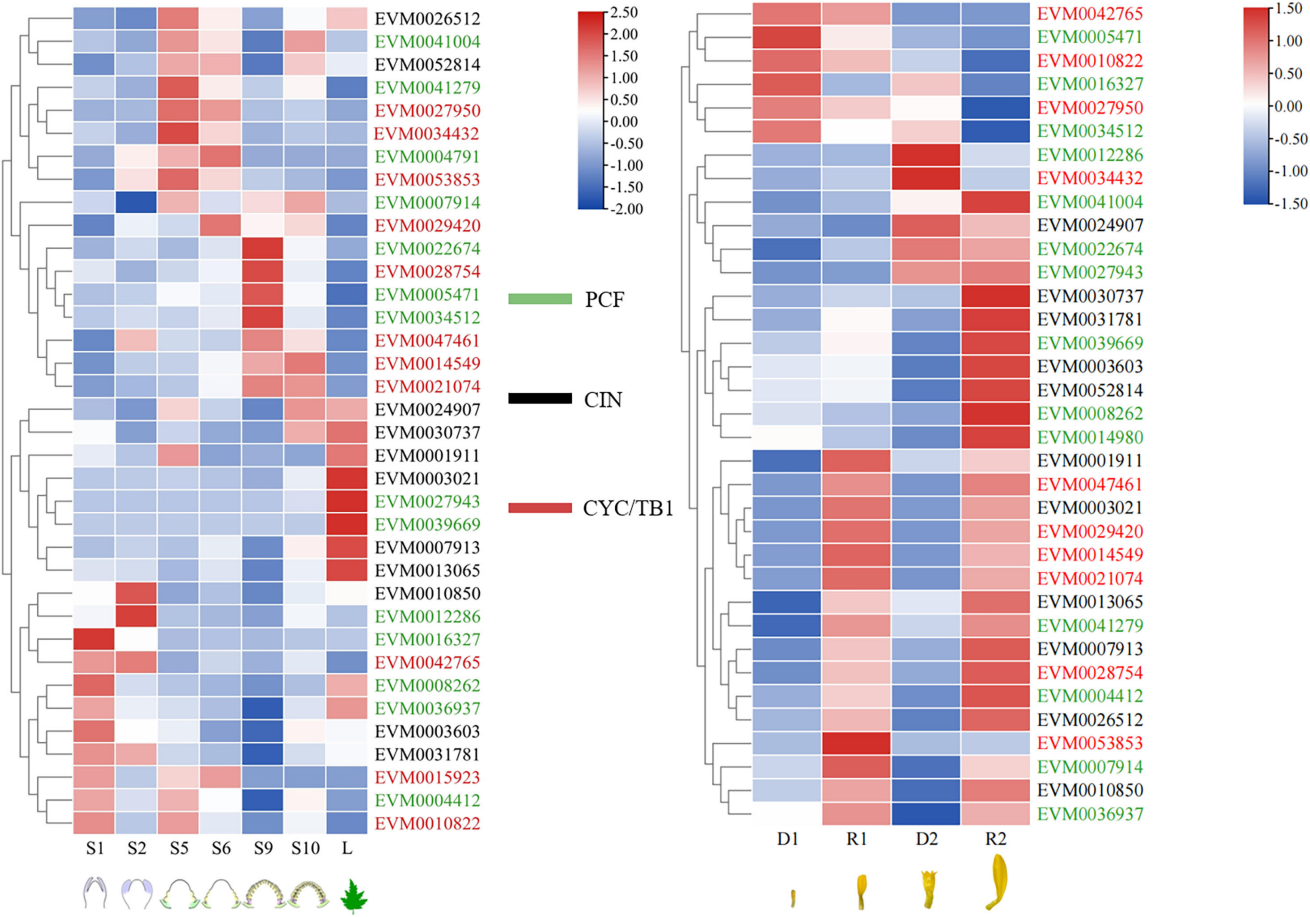
Expression patterns of *ClTCP*s during capitulum development and in two different florets of *C. lavandulifolium*. Red and blue boxes show high and low expression levels, respectively. Green, black and red color of gene names belong to PCF, CIN, and CYC/TB1, respectively. S1, S2, S5, S6, S9, and S10 indicated the different development stages of capitula (Wen et al., 2022), L indicated reproductive-stage leaf; D1, D2, R1, R2 indicated disc and ray florets at two stages.

The expression levels of *TCPs* differed in six developmental stages of capitula, 25 out of the 36 *TCP* genes were specifically expressed in flowers (**Figure 6A**). The expression levels of *EVM0029420* (*ClCYC2d*), *EVM0028754* (*ClCYC2a*), *EVM0014549* (*ClCYC2f*), and *EVM0021074* (*ClCYC2e*) in CYC/TB1 subclade in S5 stage were significantly lower than those in S6 stage, and they expressed steadily and continuously in the subsequent S9 and S10 stages. This suggested that *CYC2*-like genes in *C. lavandulifolium* were expressed at the early stage of the capitula, and ultimately played a role in the ontogenetic differences between DFs and RFs. The expression level of *EVM0034432* (*ClCYC1c*) in S5 stage was significantly upregulated compared to S6 stage and decreased continuously in S9 and S10 stages.

**Figure 6B** showed the results of hierarchical clustering to further analyze the potential involvement of *TCPs* in the development of DFs and RFs. According to their expression patterns, the *TCPs* in *C. lavandulifolium* are categorized into three clusters. The *TCPs* in *C. lavandulifolium* were grouped into three clusters based on their expression profiles. Cluster 1 consisted of the genes whose expression levels in RFs were remarkably lower than those in DFs. Conversely, the genes in Cluster 2 whose expression levels in RFs were markedly elevated compared to DFs, and Cluster 2 is the largest group. In Cluster 3, the expression levels of *ClTCPs*, such as *EVM0024907* (*ClTCP22*), *EVM0022674* (*ClTCP15*), and *EVM0027943* (*ClTCP10*) showed an increase at the late stage of two florets development. In CYC/TB1 subclade, *EVM0047461* (*ClCYC2c*), *EVM0029420* (*ClCYC2d*), *EVM0014549* (*ClCYC2f*), *EVM0021074* (*ClCYC2e*), *EVM0028754* (*ClCYC2a*) and *EVM0053853* (*ClCYC2a*) were detected to be highly expressed in RFs at two stages, but *EVM0027950* (*ClCYC1a*) was detected to be highly expressed in DFs at two stages.

### 3.6 Expression analysis of *ClCYC2* genes in different *Chrysanthemum* species

**Figure 7:**
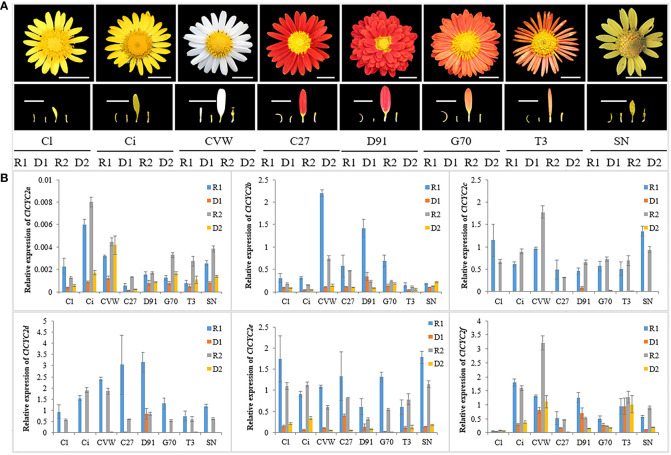
Relative expression levels of *ClCYC* genes in eight different *Chrysanthemum* samples using qRT-PCR. **(A)** Capitulum and DFs and RFs of two development stages of *C. lavandulifolium* (Cl), *C. indicum* (Ci), *C. vesticum* (CVW), *C. ×morifolium* ‘C27’, ‘D91’, ‘G70’, ‘T3’ and *C. aromaticum* (SN). Bars=1 cm. **(B)** Expression levels of six *ClCYC2* genes in DFs and RFs of the above samples. Data were normalized to the expression data of *ClSAND* and expressed as the means ± standard error of three biological replicates.

To verify the RNA-seq data and determine *ClCYC2* genes, which belong to the class II TCP subclade, and might be responsible for the development of RFs and DFs, qRT-PCR was executed to assess their expression profiles in RFs and DFs during capitulum development. Notably, six *ClCYC2* genes were differentially expressed between RFs and DFs at two developmental stages. Almost all of these genes were highly expressed in the RFs of *C. lavandulifolium*, whereas they showed almost no or very low expression levels in the DFs, which was similar to previous research (Huang et al., 2016; Chen et al., 2018; Wen et al., 2019a). Among them, the expression difference between *ClCYC2c* and *ClCYC2d* in the two florets was the most significant (**Figure 7**).

Next, other seven *Chrysanthemum* samples from the chrysanthemum germplasm nursery (**Figure 7**) were used to determine whether these *CYC2*-like exhibited similar expression patterns in the RFs and DFs of *Chrysanthemum* species. Notably, the expression patterns of *CYC2* genes between two florets of different *Chrysanthemum* samples were almost similar, which suggested that the functions of the *CYC2* gene may be conserved in the development of two florets of *Chrysanthemum*. In conclusion, we regarded *ClCYC2c* and *ClCYC2d* as the key genes responsible for DFs and RFs development in *C. lavandulifolium*.

### 3.7 Subcellular localization and transactivation activity of ClCYC2 proteins

**Figure 8:**
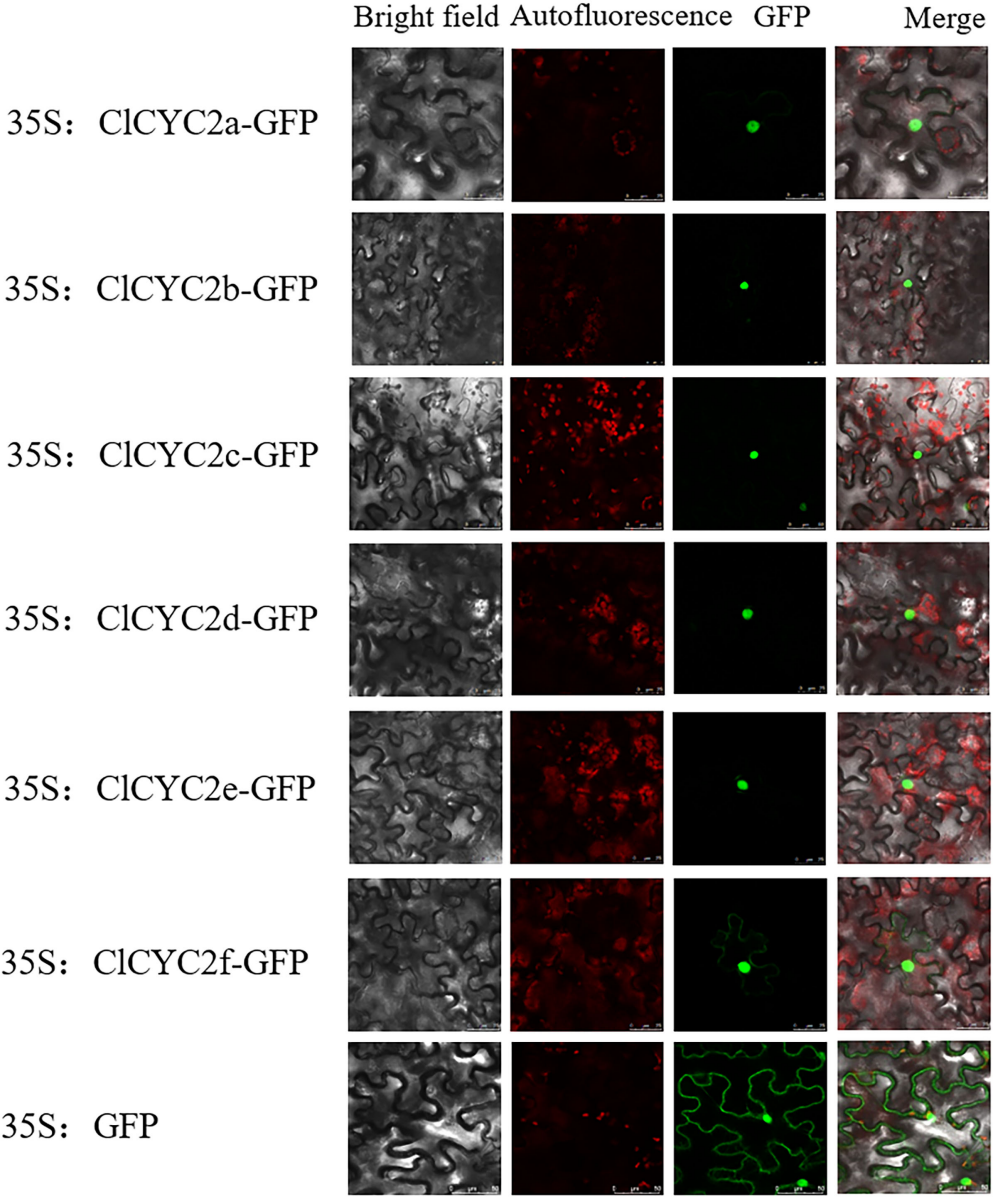
Subcellular localization of six ClCYC2 proteins. Six ClCYC2-GFP fusion recombinants (ClCYC2a-GFP, ClCYC2b-GFP, ClCYC2c-GFP, ClCYC2d-GFP, ClCYC2e-GFP, and ClCYC2f-GFP) and pBI121-GFP as control were transiently expressed in *N. benthamiana* leaves and observed by a laser scanning confocal microscope 48 hours after injection. Bar=25 µm.

Generally, transcription factors (TFs) can regulate the candidate genes by interacting with specific *cis*-regulatory elements located in their promoter regions, which occur in the nucleus. In this study, according to WoLF PSORT predictions, most ClTCP proteins were anticipated to be located in the nucleus (**Table 1**). To examine this feature, the CDSs of the *ClCYC2a*, *ClCYC2b*, *ClCYC2c*, *ClCYC2d*, *ClCYC2e*, and *ClCYC2f* without stop codons were amplified with the primer pairs (**Supplemental Table 2**) and recombined into pBI121-eGFP to generate six fusion constructs, then the fusion proteins and pBI121-eGFP were expressed transiently in *N. benthamiana* leaves via Agrobacterium-regulated transformation. The results showed that the control vector pBI121-eGFP had a strong fluorescence signal in the whole cell, and the distribution of the fluorescence signal in the cell was not specific. The fluorescence signals of the six ClCYC2 vectors were localized in the nucleus (**Figure 8**), which was similar to the results in Moso Bamboo (Liu et al., 2018), *Vitis vinifera* (Jiu et al., 2019) and *Ipomoea batatas* (Ren et al., 2021). The positive controls for each test to ensure the reliability of the results. These results indicated that six ClCYC2 proteins of *C. lavandulifolium* were nuclear proteins, and consistent with the general characteristic of nuclear localization of TFs.

**Figure 9:**
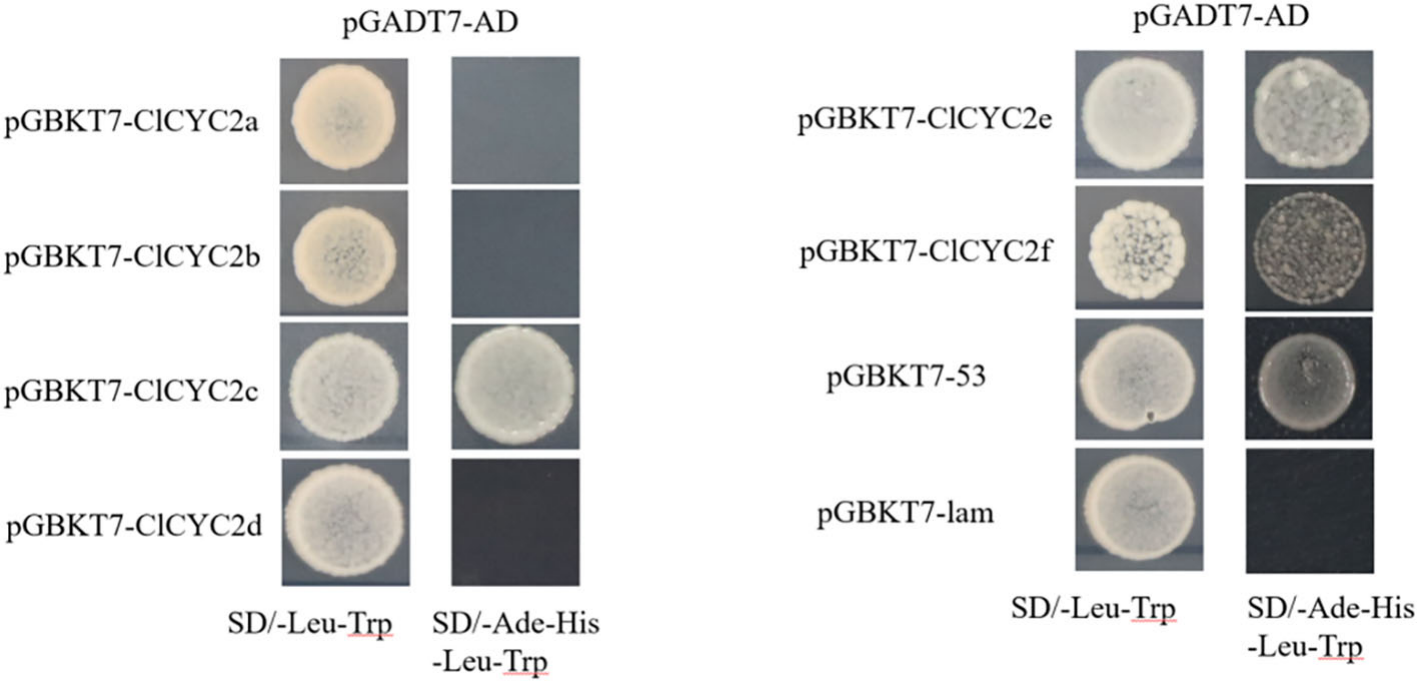
Transactivational analysis of ClCYC2 proteins in Y2HGold strain. The fusion constructs with pGADT7 AD empty, positive constructs (pGBKT7-53) and negative constructs (pGBKT7-lam) were co-transformed into Y2HGold strain and incubated on the SD/-Leu-Trp and SD/-Ade-His-Leu-Trp media.

To explore the transactivational activity levels of six ClCYC2 proteins, the pGBKT7-ClCYC2 recombinant vectors (gene-specific primer pairs in **Supplementary Table 3**), the pGBKT7-53 (positive control) and pGBKT7-lam with pGADT7 empty vectors (negative control) were co-transformed into theY2HGold strain, respectively. All of these transformants grew well and exhibited obvious white colonies on the SD/-Leu-Trp medium (**Figure 9**). Only the yeast cells carrying *ClCYC2c*, *ClCYC2e*, *ClCYC2f*, and the positive group could grow well on the SD/-Ade-His-Leu-Trp medium. Whereas, the yeast cells harboring *ClCYC2a*, *ClCYC2b*, *ClCYC2d*, and the negative group did not proliferate (**Figure 9**). Hence, three out of six candidate Class II *ClTCP* constructs activated the levels of the *His3* and *Ade2* reporter genes, demonstrating their transactivational activities in the yeast strain. To screen the interacting proteins in the subsequent yeast two-hybridization system, the bait expression vectors with self-activating activities were inhibited by adding 3-AT. The results showed that the bait vectors of ClCYC2c, ClCYC2e, and ClCYC2f could inhibit their self-activating activities by adding 80 mM, 20 mM, and 10 mM 3-AT, respectively.

## 4 Discussion

TCP protein is a TF specific to plants, which is involved in different aspects of biological and physiological processes throughout plant growth/development (Manassero et al., 2013). *TCPs* have yet been discovered and characterized in various plants, including *Arabidopsis* (Riechmann et al., 2000; Li, 2015), *M. domestica* (Xu et al., 2014), Legume (Ling et al., 2020), *Vitis vinifera* (Jiu et al., 2019), *P. mume* (Zhou et al., 2016), Moso Bamboo (Liu et al., 2018) and *C. nankingense* (Tian et al., 2022). Nonetheless, no comprehensive analysis of *TCPs* in *C. lavandulifolium*, a prominent ancestral species of cultivated chrysanthemum has been undertaken. In the study, 40 *ClTCPs* were determined from *C. lavandulifolium* genome and transcriptome. Moreover, we performed a comprehensive analysis of the *ClTCPs* by assessing their phylogenetic relationships, chromosomal position, conserved motifs, gene structures, promoter analysis, expression patterns in different developmental stages and different flower types, and subcellular localization and transactivation activity of key ClTCP proteins. These findings offer a basis for further investigations on the functions of *ClTCPs* during flowering.

### 4.1 Evolutionary conservation and divergence of *TCPs* in *C. lavandulifolium*

In *C. lavandulifolium*, 40 *TCPs* were discovered in the genome and transcriptome, and their distributions on the chromosomes were not uniform. The number of ClTCPs in *C. lavandulifolium* is about double that of *C. nankingense*, *V. vinifera*, and *Arabidopsis*, which possess 23, 17, and 24 TCP members, respectively (Li, 2015; Jiu et al., 2019; Tian et al., 2022). The collinearity analysis of the *V. vinifera* and *C. lavandulifolium* genomes revealed a 1 *vs*. 3 syntenic association between them, providing compelling evidence for whole-genome triplication (WGT-1) in *C. lavandulifolium* (Wen et al., 2022). The WGT event was shared by Asteraceae species and may be responsible for the emergence of complex traits in the capitulum. Earlier research has established that *CYC2* clade genes play a crucial role in regulating DFs and RFs development in Asteraceae species, including *G. hybrida*, *H. annuus*, *S. vulgaris*, and *C. ×morifolium* (Tahtiharju et al., 2012; Garces et al., 2016; Huang et al., 2016; Chen et al., 2018). We identified nine *CYC2*-like genes in *C. lavandulifolium* (**Figure 3**). It possesses a larger number of *TCPs*, amounting to nine members, which surpasses the 7 and 8 members identified in *C. nankingense* and *H. annuus*, respectively, but fewer than 25 members found in *C.* ×*morifolium* (Song et al., 2023). Besides, tandem duplication events occurred in the evolutionary process in *C. lavandulifolium CYC2* genes, which led to *CYC2a* multicopy genes forming a gene cluster on chromosome 6. The duplication event likely facilitated the proliferation of *CYC2c/2d/2e/2f*, and subsequent evolutionary processes could have led to subfunctionalization of these genes within the *C. lavandulifolium* genome (**Figure 2**). The notable difference in the number of *TCPs* between *C. lavandulifolium* and *C. nankingense* might be due to the divergency time, that *C. lavandulifolium* and *C. nankingense* diverged from each other around 7.2 Mya (Wen et al., 2022). The previous result shows the class II *TCPs* in *C. lavandulifolium* were significantly expanded (**Figure 3**).

According to multiple sequence alignments and phylogenetic analyses, all 40 *ClTCPs* were grouped into 3 subgroups (**Figures 1** and **3**), which was in accordance with those in *Arabidopsis* (Li, 2015). Generally, *TCPs* within a clade have similar gene structures, chromosomal locations and motif distributions, suggesting that *TCPs* are evolutionarily conserved. For example, we found the highly-conserved TCP domains (motif 1) occurred in all ClTCP proteins (**Figure 4**), while motif 2 was only present in the class I PCF clade and motif 7 was detected in most of class II CIN proteins (**Figure 4B**). The close evolutionary relationships are further supported by the fact that the motif compositions and gene structures of *ClTCPs* exhibit a high degree of consistency. Additionally, our analysis revealed the presence of an arginine-rich R domain at the C-terminus of all ClTCP proteins classified under class II CYC/TB1, and one member (EVM0024660) from CIN. R domain was estimated to increase protein-protein interactions, as do four members (AtTCP2, 12, 18, and 24) in *Arabidopsis.*

Subcellular localization indicated that six ClCYC2 proteins of *C. lavandulifolium* were nuclear proteins, but these six CYC2 members have different transcriptional activation activities, which may be affected by several factors, including the binding ability of the TCP transcription factors to the target genes, differences in gene structures and sequences, differences in expression regulatory mechanisms, and the involvement of other cofactors (Martin-Trillo et al., 2011; Tahtiharju et al., 2012; Valsecchi et al., 2013; Parapunova et al., 2014). Further exploration and experimental verification will help to fully understand the regulatory mechanisms of these key *ClCYC2* genes.

### 4.2 The potential roles of *ClTCP*s in plant growth and development

The function of a gene is closely correlated with its expression patterns (Xu et al., 2015). Herein, we determined the expression patterns of all *ClTCPs* during flower development by RNA-seq data reported previously (Wen et al., 2019a; Wen et al., 2022) and detailed expression patterns of key class II *ClTCPs* (*ClCYC2*) in different *Chrysanthemum* samples via real-time RT-PCR. The result indicated that these genes exhibited unique expression patterns during capitulum development and in different flower types. In detail, most *ClTCPs* were specifically expressed in flowers during capitulum development, and *TCPs* were categorized into three clusters in accordance with their expression patterns in DFs and RFs. The expression levels of *CYC2* genes in RFs were upregulated compared to DFs, but the expression profiles of *CYC1* and *CYC3* group genes were different from those of *CYC2* genes in *C. lavandulifolium*, which presumed that they had different functions during flower development. These suggested that *TCPs* in *C. lavandulifolium* were associated with the growth of capitula or two florets.

In earlier research (Sarvepalli and Nath, 2018), the CIN subclade *TCPs* were found to primarily regulate leaf development, floral organ development, and flowering time. In Arabidopsis, 5 CIN-like genes have been reported to undergo post-transcriptional regulation by microRNA319 (miR-319). Notably, the regulation of TCP4 by miR-319a is crucial for the development of stamens and petals (Nag et al., 2009). In *C. lavandulifolium*, four homologs of CIN subclade genes (*ClTCP24*, *ClTCP25*, *ClTCP26* and *ClTCP27*) included the potential binding sites for miR319 (**Figure 1C**). *ClTCP24*, *ClTCP25* and *ClTCP26* are upregulated in leaves, suggesting their potential roles in leaf development. The conservation of miR319 targeting may extend to other *TCPs* as well (Palatnik et al., 2003; Seki et al., 2020).

Moreover, promoter analysis shows that the promoter region of *ClTCPs* was found to contain a substantial number of hormone-responsive elements (**Figure 5**), implying these genes might exert significant influences on hormone-responsive plant developmental processes. Previous reports have shown that TFs and phytohormones play important roles in determining flower size in *Arabidopsis* (Krizek and Anderson, 2013). Cytokinin has been demonstrated to influence the duration of cell division in developing floral organs (Bartrina et al., 2011). Considering the abundant hormone-responsive elements distributed in *ClTCP* gene promoters (**Figure 5**), we speculate that *ClTCPs* may serve as important regulators of hormone-induced alterations in plant development. Of course, this hypothesis needs to be further tested.

### 4.3 Contribution of *CYC2* genes to the morphological differences of two florets in *Chrysanthemum*

Flower development in the angiosperm is a complex physiological and morphological process, which was determined by a set of TFs regulatory networks (Soltis et al., 2002). Much evidence has already suggested that *TCPs*, especially the *CYC2* clade genes affect the morphology of capitula by regulating the reproductive organ development, inflorescence architecture and floral symmetry (Juntheikki-Palovaara et al., 2014; Huang et al., 2016; Chen et al., 2018; Elomaa et al., 2018).

Our investigation revealed the presence of 13 genes in *C. lavandulifolium* that belong to the CYC/TB1 subclade, CYC2 group has nine members with the largest number. Among them, six *TCPs* from CYC2 group, *EVM0047461* (*ClCYC2c*), *EVM0029420* (*ClCYC2d*), *EVM0014549* (*ClCYC2f*), *EVM0021074* (*ClCYC2e*), *EVM0028754* (*ClCYC2a*) and KX161380.1 (*ClCYC2b*) were all highly expressed in the RFs, whereas they shown almost no or very low expression levels in DFs (**Figure 7**). And the difference in expression was universal in different *Chrysanthemum* species, which suggested the functions of the *CYC2* gene may be conserved in the two florets development of *Chrysanthemum*, whereas certain members of the CYC/TB1 subclade experienced functional divergence subsequent to gene duplication events. Based on previous studies, it was hypothesized that *CYC2c* and *CYC2d* are mainly involved in RF identity and thus affect the development of capitulum, *CYC2b* and *CYC2e* tend to regulate petal length in RFs (Shen et al., 2021), and CYC2c can interact with the *CYC2f* promoter to modulate floral symmetry development (Yuan et al., 2020). Moreover, six *CYC2* genes were expressed in *C. lavandulifolium* at the early capitulum developmental stages, which was consistent with the results of nucleic acid *in situ* hybridization of *Gerbera* and *S. vulgaris* that detected *CYC2*-like gene expression in the meristem and floret primordium at the early capitula development (Garces et al., 2016; Elomaa et al., 2018). This implied that *CYC2* genes in *C. lavandulifolium* were expressed at the early stages of the capitula, and ultimately played roles in the ontogenetic differences between ray and disc florets.

## 5 Conclusion

In summary, the first genome-wide analyses of *TCPs* in *C. lavandulifolium* were conducted. 40 *ClTCPs* were discovered and distributed on 8 chromosomes. These *ClTCPs* were classified into two main groups in accordance with the phylogenetic analysis and structural characteristics. The *ClTCP* promoter sequences had many different kinds of *cis*-acting elements, suggesting that *ClTCPs* were controlled by a complex regulatory network. *ClTCPs*, especially those from class II CYC/TB1 clade, may be crucial for the two florets development in *Chrysanthemum* as indicated by their expression patterns and previous studies. Notably, six candidate *ClCYC2* genes were higher expressed in RFs than those in DFs, which speculated that the *CYC2* genes may be functionally conserved in two florets development in *Chrysanthemum*. The subcellular localization and transactivation activity analysis of six candidate TCP proteins were also examined in *C. lavandulifolium*. In short, our study increased the understanding of *ClTCP* functions during flower development in *Chrysanthemum* species. Based on this study, in the future we can validate the functions of key *TCPs* in native plants, and deeply study the interactions between the key *ClTCP*s and their upstream and downstream regulatory mechanisms, to resolve the molecular network of *TCPs* involved in the capitulum development of *Chrysanthemum*.

## Data availability statement

The original contributions presented in the study are included in the article/**Supplementary Material**. Further inquiries can be directed to the corresponding author.

## Author contributions

XWu: Conceptualization, Data curation, Formal Analysis, Investigation, Methodology, Resources, Software, Validation, Visualization, Writing – original draft, Writing – review & editing. JL: Resources, Software, Writing – review & editing. XWe: Resources, Writing – review & editing. QZ: Writing – review & editing. SD: Conceptualization, Funding acquisition, Project administration, Supervision, Validation, Writing – review & editing.
